# The effects of spatial population dataset choice on estimates of population at risk of disease

**DOI:** 10.1186/1478-7954-9-4

**Published:** 2011-02-07

**Authors:** Andrew J Tatem, Nicholas Campiz, Peter W Gething, Robert W Snow, Catherine Linard

**Affiliations:** 1 Department of Geography, University of Florida, Gainesville, USA; 2 Emerging Pathogens Institute, University of Florida, Gainesville, USA; 3 Fogarty International Center, National Institutes of Health, Bethesda, MD 20892, USA; 4 Spatial Ecology and Epidemiology Group, Tinbergen Building, Department of Zoology, University of Oxford, South Parks Road, Oxford, UK; 5 Malaria Public Health and Epidemiology Group, Centre for Geographic Medicine, KEMRI - University of Oxford - Wellcome Trust Research Programme, Nairobi, Kenya; 6 Centre for Tropical Medicine, Nuffield Department of Clinical Medicine, University of Oxford, CCVTM, Oxford, UK; 7 Biological Control and Spatial Ecology, Université Libre de Bruxelles, Brussels, Belgium

## Abstract

**Background:**

The spatial modeling of infectious disease distributions and dynamics is increasingly being undertaken for health services planning and disease control monitoring, implementation, and evaluation. Where risks are heterogeneous in space or dependent on person-to-person transmission, spatial data on human population distributions are required to estimate infectious disease risks, burdens, and dynamics. Several different modeled human population distribution datasets are available and widely used, but the disparities among them and the implications for enumerating disease burdens and populations at risk have not been considered systematically. Here, we quantify some of these effects using global estimates of populations at risk (PAR) of *P. falciparum *malaria as an example.

**Methods:**

The recent construction of a global map of *P. falciparum *malaria endemicity enabled the testing of different gridded population datasets for providing estimates of PAR by endemicity class. The estimated population numbers within each class were calculated for each country using four different global gridded human population datasets: GRUMP (~1 km spatial resolution), LandScan (~1 km), UNEP Global Population Databases (~5 km), and GPW3 (~5 km). More detailed assessments of PAR variation and accuracy were conducted for three African countries where census data were available at a higher administrative-unit level than used by any of the four gridded population datasets.

**Results:**

The estimates of PAR based on the datasets varied by more than 10 million people for some countries, even accounting for the fact that estimates of population totals made by different agencies are used to correct national totals in these datasets and can vary by more than 5% for many low-income countries. In many cases, these variations in PAR estimates comprised more than 10% of the total national population. The detailed country-level assessments suggested that none of the datasets was consistently more accurate than the others in estimating PAR. The sizes of such differences among modeled human populations were related to variations in the methods, input resolution, and date of the census data underlying each dataset. Data quality varied from country to country within the spatial population datasets.

**Conclusions:**

Detailed, highly spatially resolved human population data are an essential resource for planning health service delivery for disease control, for the spatial modeling of epidemics, and for decision-making processes related to public health. However, our results highlight that for the low-income regions of the world where disease burden is greatest, existing datasets display substantial variations in estimated population distributions, resulting in uncertainty in disease assessments that utilize them. Increased efforts are required to gather contemporary and spatially detailed demographic data to reduce this uncertainty, particularly in Africa, and to develop population distribution modeling methods that match the rigor, sophistication, and ability to handle uncertainty of contemporary disease mapping and spread modeling. In the meantime, studies that utilize a particular spatial population dataset need to acknowledge the uncertainties inherent within them and consider how the methods and data that comprise each will affect conclusions.

## Background

Defining the extent of infectious diseases as a public health burden and their distribution and dynamics in time and space are critical to disease monitoring, control, and decision-making. The epidemiology of many diseases makes surveillance-based methods for estimating populations at risk and disease burden problematic [1-3], while spatial heterogeneity in human population distribution can produce significant effects on transmission [4,5]. Cartographic and spatial modeling approaches have proven to be effective in tackling these factors [6-8]. Such approaches can help characterize large-scale patterns of disease spread to evaluate intervention impact [4] and produce globally consistent measures of morbidity of known fidelity, often the only plausible method in many African countries where surveillance data are incomplete, unreliable, and inconsistent [1,9,10]. However, any approach that requires the use of modeled disease rates or dynamics to estimate risk requires reasonable information on the distributions of resident populations. Where risks and the spread of diseases are heterogeneous in space, population distributions and counts must be resolved to reasonably high levels of spatial detail.

National census population data have often been represented as continuous gridded population distribution (or count) datasets through the use of spatial interpolation algorithms. Four differing approaches to the interpolation of census data have been used to create four different global population distribution databases at spatial resolutions of finer than 1 degree, each of which has been used in epidemiological studies. These are LandScan [11], the Gridded Population of the World (GPW) [12], the Global Rural Urban Mapping Project (GRUMP) [13], and the United Nations Environment Programme (UNEP) Global Population Databases [14]. Features of each dataset are outlined in Table 1, their full extents are mapped in Additional file 1, Figure S1, and each is discussed in more detail below.

**Table 1:** Gridded population datasets and their characteristics.

Dataset	Year(s) represented	Spatial resolution	Input data used	Data source for national pop total adjustments	Source
LandScan	2008	30 arcseconds (~1 km)	Census, land cover, elevation, slope, roads, populated areas/points	CIA [23]	[11]; http://www.ornl.gov/sci/landscan/

Gridded Population of the World (GPW)	1990/1995/2000/2005/2010/2015	2.5 arcminutes (~5 km)	Census, water bodies (for masking)	UNPD [22]	[12]; http://sedac.ciesin.columbia.edu/gpw/global.jsp

Global Rural Urban Mapping Project (GRUMP)	1990/1995/2000	30 arcseconds (~1 km)	Census, populated areas, water bodies (for masking)	UNPD [22]	[13]; http://sedac.ciesin.columbia.edu/gpw/global.jsp

United Nations Environment Programme (UNEP) Global Population Databases	2000	2.5 arcminutes (~5 km)	Census, populated points, roads	UNPD [22]	[14]; http://na.unep.net/siouxfalls/datasets/datalist.php

**Figure 1:**
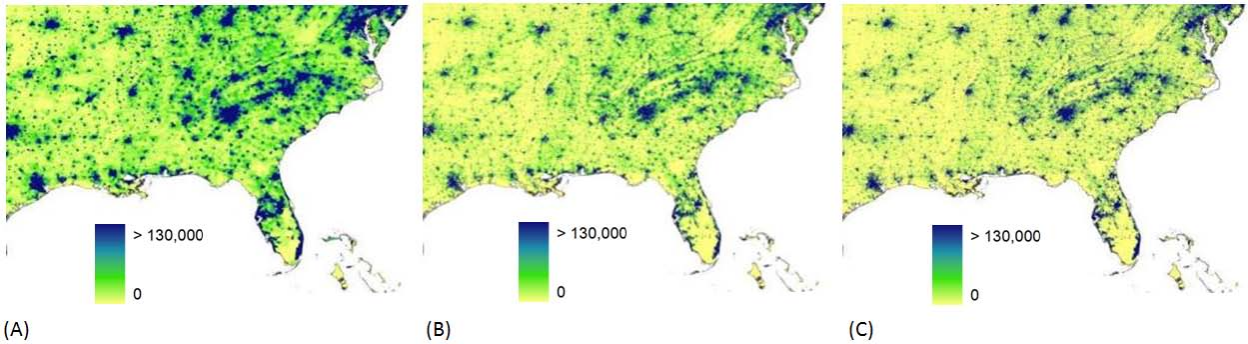
**Population distribution for the southeast United States as mapped by three different datasets**. The datasets shown are (A) GPW (B) GRUMP (C) LandScan. The UNEP Global Population Databases product is not represented here as it does not cover the USA. The values shown are estimates of persons per grid square.

**Figure 2:**
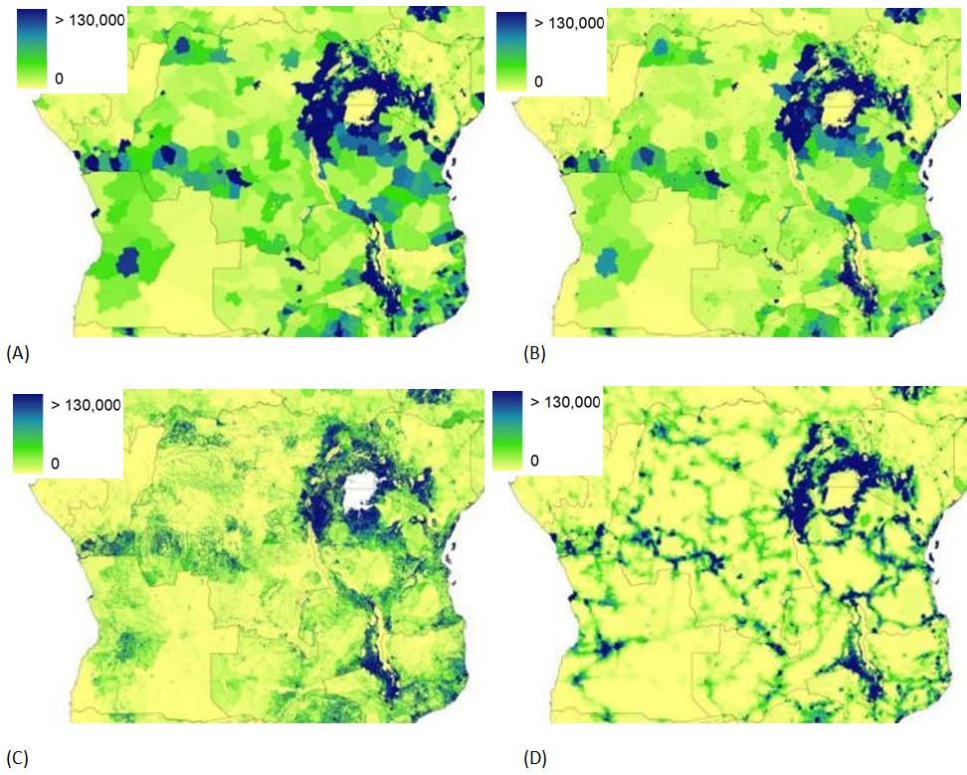
**Population distribution for central and east Africa as mapped by four different datasets**. The datasets shown are (A) GPW (B) GRUMP (C) LandScan (D) UNEP, and the values shown are estimates of persons per grid square.

Population census data are the core inputs to spatial population databases and, for many countries, contemporary census data collected at a high administrative-unit level exist to facilitate detailed and precise population mapping. For the majority of low-income countries of the world, however, spatially detailed, contemporary census data to facilitate such detailed mapping do not exist. This is especially true for much of Africa. Census data used for the production of global products are more than a decade old in 38 of 56 African countries and, at administrative boundary levels, just one or two levels finer than national level in 44 countries [15]. The poor quality of the inputs propagates differently through the four modeled human population distributions, as contrasted by maps of the different distributions from the southeastern United States (Figure 1) and for central Africa (Figure 2). The population distributions for the southeastern United States quantified by the GPW, GRUMP, and LandScan datasets appear very similar, where highly resolved census tract-level count data provide the main input. Such detailed representations often prompt misconceptions that population distribution is now known and mapped accurately for the entire world [4,16]. The same population density datasets for central Africa highlight the differences, however, where input census data vary substantially in quality (Figure 2). The differing approaches to the spatial interpolation of poorly resolved census data produce very different spatial configurations of population.

Each of the four spatial population datasets has been used extensively in epidemiological studies during the past two decades (Table 2). Different authors have used different population datasets for the same purpose, yet the accuracies, variations, and effects upon results that this choice entails have yet to be examined. Applications have involved estimating numbers of clinical cases, spread modeling, risk mapping, quantifying the effects of urbanization, and studying diseases ranging from dengue and yellow fever to HIV and leprosy. The most widespread use of gridded population datasets in an epidemiological context has been in the study of malaria (Table 2) with a variety of purposes (Additional file 1, Table S1). All four global datasets used to derive estimates have been used to estimate populations at risk (PAR) of malaria, forming a fundamental metric for decision-makers at national and international levels [9,17]. Here, to illustrate the effects of spatial population dataset choice in an applied epidemiological setting, we undertake a set of analyses to quantify the spatial variation and sizes of absolute and relative differences in PAR of *P. falciparum *malaria that can be obtained through the use of differing population datasets. We then discuss how these differences arise, their likely translation to other disease systems, and approaches to dealing with the uncertainties in large-scale spatial population datasets.

**Table 2:** Disease-related studies that have utilized large area gridded population datasets.

Disease	Application	Population map used [Reference]
Malaria	Populations at risk	GPW [47-52], LandScan [53,54], UNEP [55], GRUMP [24,25,28,30-32,56-58]
	Clinical cases	GPW [59], GRUMP [8]
	Intervention coverage	GRUMP [60]
	Funding coverage	GRUMP [57,61]
	Risk mapping	GPW [62-64], UNEP [65], GRUMP [66]
	Infection movements	GRUMP [29,30]
	Urbanization effects	GPW [67], GRUMP [15]

Hookworm	Populations at risk	GPW [68,69]

Influenza	Epidemic modeling	GPW [70-73], LandScan [7,74,75], GRUMP [76]
	Risk mapping	LandScan [77,78]

Yellow fever	Populations at risk	GRUMP [79]

Dengue	Populations at risk	GRUMP [79]
	Risk mapping	USGS [21], LandScan [80,81]

Filariasis	Populations at risk	UNEP [82]

Helminths	Populations at risk	GPW [52,83], UNEP[84]

Bovine TB	Risk mapping	LandScan [85,86], GPW [86]

Trypanosomiasis	Risk mapping	UNEP [87]

Onchocerciasis	Populations at risk	GPW [88]

Leprosy	Risk mapping	GPW [89]

HIV	Prevalence analyses	LandScan [90]

General	Trends in emerging diseases	GPW [91]
	Health of schoolchildren	UNEP [92]

GPW = Gridded Population of the World, GRUMP = Global Rural Urban Mapping Project, UNEP = United Nations Environment Programme Global Population Databases, USGS = United States Geological Survey Population datasets.

## Methods

Assessment of the effects of spatial population dataset choice on estimates of populations at risk of *P. falciparum *is undertaken here through three steps: (i) gathering existing spatial population datasets; (ii) overlaying *P. falciparum *transmission maps onto each population dataset, extracting populations at risk and quantifying the range of estimates achievable; (iii) and assessing which population modeling method results in more accurate estimates of populations at risk in three test countries where population distribution is known with greater precision than the input data used in construction of the datasets being tested. The datasets and methods used for each of these steps are described in detail in the following sections.

### Global spatial population datasets

Analyses here focus on the four datasets most commonly used in disease-related studies, and principally on LandScan and GRUMP, the most contemporary and widely used datasets (Table 2). These two datasets have become more widely used in epidemiology due to their finer spatial resolution than GPW and UNEP, the fact that UNEP has not been updated for more than a decade, and the inclusion of urban extents in GRUMP that improves mapping precision over GPW [18]. Inputs to and outputs of the four datasets differ (Table 1, Figures 1-2). We do not consider here coarse datasets (1 degree spatial resolution or coarser), such as that outlined by Li et al [19], that have occasionally been used in disease-related studies [20,21]. Table 1 provides references and Web links for detail on each spatial population dataset, and each is shown in Additional file 1, Figure S1.

In constructing the global population datasets, the use of census counts provided by national statistics offices and resulting intercensal growth rates lead to a patchwork of datasets, methods, and total national counts that are different from widely used and standardized estimates made by international agencies [22,23]. Thus, each product is adjusted to match national totals estimated by one of these agencies for the product year in question. LandScan adjusts its totals to match those estimates made by the Central Intelligence Agency (CIA)[23], while the remaining datasets adjust to the United Nations Population Division (UNPD) estimates [22]. Differences in estimates made by these different agencies translate into differences in PAR, numbers in susceptible, infected, and recovered model groups, and many other epidemiological measures. Initially, therefore, 2010 national population estimates made by the CIA and UNPD were obtained and the differences explored.

### Assessing variations in global PAR of *P. falciparum* malaria

**Figure 3:**
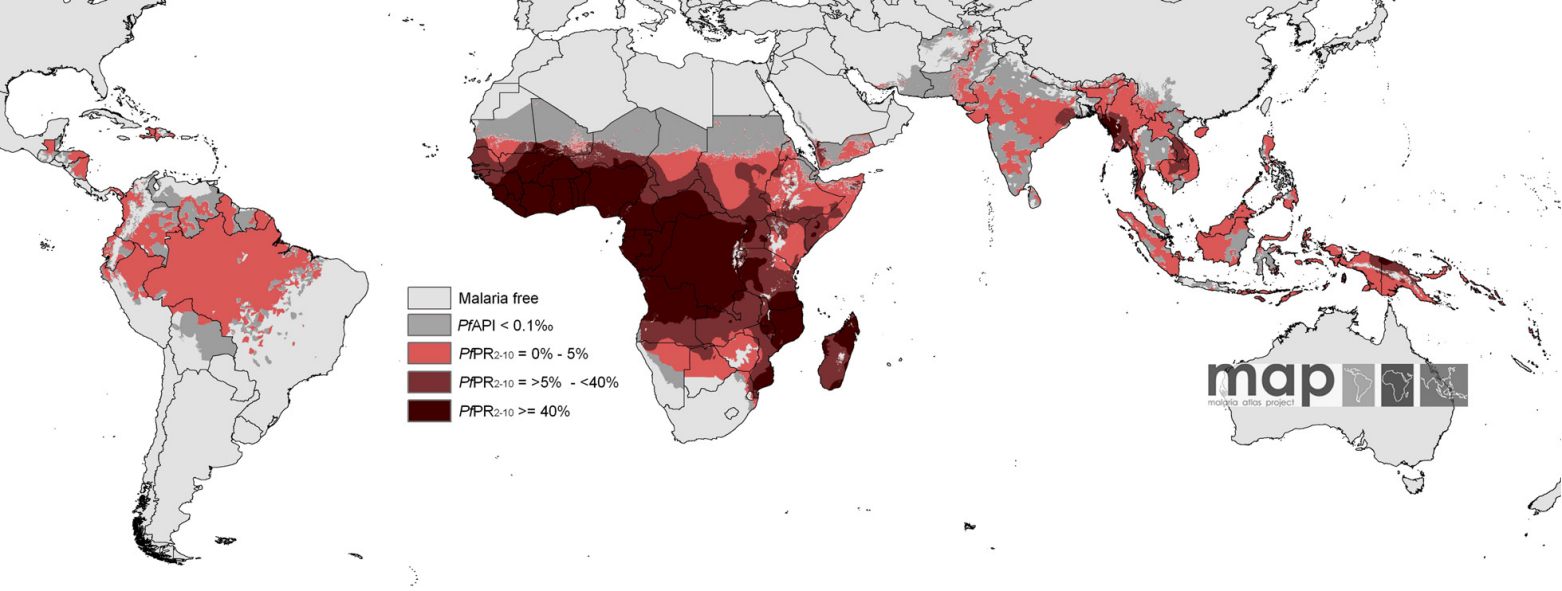
**The spatial distribution of *P. falciparum *malaria *Pf*PR2-10 predictions stratified by endemicity class**. They are categorized as low risk *Pf*PR2-10 <5%, light red; intermediate risk *Pf*PR2-10 = 5% to 40%, medium red; and high risk *Pf*PR2-10 >40%, dark red. The map shows the class to which *Pf*PR2-10 has the highest predicted probability of membership. The rest of the land area was defined as unstable risk (medium gray areas, where *Pf*API = 0.1 per 1,000 pa) or no risk (light gray).

The Malaria Atlas Project has recently published revised global limits of unstable and stable *P. falciparum *infection risk [24] and a modeled, mapped distribution of the intensity of *P. falciparum *within the stable margins of transmission based upon infection prevalence among children aged 2 to 10 years (*Pf*PR_2-10_) [25]. In brief, data on national case reporting, national and international medical intelligence, climate, and aridity were used to define conservatively the margins of stable and unstable *P. falciparum *transmission [24]. Stable malaria transmission was assumed to represent a minimum average of 1 clinical case per 10,000 population per annum (pa) in a given administrative unit. Unstable malaria transmission was used to define areas where transmission was biologically plausible and/or had been documented but where incidence was likely to be less than 1 case per 10,000 population pa. In Africa, this was largely in areas where aridity limits the survival of larvae and causes desiccation of adult vectors. Finally, no transmission was assumed where assembled intelligence stated no malaria risk because (1) national reporting systems had, over several years, not reported a single *P. falciparum *clinical case, or (2) where temperatures were too low for sporogony to complete within the average lifespan of the local dominant vector species. Within the stable transmission margins, empirical community survey data on parasite prevalence were assembled and geolocated to provide the basis for an urban-rural and sample-size-adjusted geospatial model within a Bayesian framework to interpolate a continuous space-time posterior prediction of *Pf*PR_2-10 _for every 5 × 5 km pixel for the year 2007 [25]. This model also generated classified output that assigned each pixel to one of four malaria endemicity classes: malaria-free or unstable, *Pf*PR_2-10 _<5%; *Pf*PR_2-10 _= 5% to 40%; and *Pf*PR_2-10 _>40% (Figure 3). These classifications of stable transmission correspond to ranges of *Pf*PR that have been proposed in the selection of suites of interventions at scale to reach control targets at different time periods [26,27].

The transmission classes mapped in Figure 3 have been used in previous studies to estimate PAR using the GRUMP dataset [8,25,28-30]. Here, we examine the differences that can be obtained using alternative population datasets (Table 1). Though there exist more appropriate measures for calculating PAR that are consistent with the *P. falciparum *malaria endemicity surface and that integrate the uncertainty inherent in the *Pf*PR_2-10 _estimates [31], here we compare geographical information system (GIS) overlays as done by the vast majority of previous studies (Table 2).

We obtained the population count dataset (Table 1) closest in time, at the time of writing, to 2007, the year represented by the *P. falciparum *endemicity class map. For LandScan, this was the 2007 version. For GPW3, this was the 2005 version. For GRUMP, this was the 2000 beta version. And for UNEP, this was the 2000 product. GPW3, GRUMP, and UNEP were thus projected forward to 2007, applying national, medium variant, intercensal growth rates by country [22], using methods described previously [18], and undertaken in many previous PAR estimation studies [8,18,24,25,28-32]. The *Pf*PR_2-10 _transmission classes were overlaid onto the four population datasets, and per-country PARs for each class were extracted for analysis.

As described above, the population datasets outlined in Table 1 adjust their national totals to estimates made by differing agencies. Thus, differences in PAR estimates reflect both these adjustments to differing totals, as well as differences in the census unit disaggregation methods. To isolate and examine the effect of different disaggregation methods, population totals were linearly adjusted to common totals (in this case, those defined by the UNPD [22]) maintaining the endemicity class proportions extracted. Thus, two sets of analyses were undertaken: those that examined PAR differences based on the unadjusted native products, as undertaken in epidemiological studies to date (Table 2), and those that examined PAR differences based on adjusting national populations to a common total to examine the effect of differing census data disaggregation approaches.

### National-level assessments of PAR estimates

Validation and accuracy assessment of high-resolution population data is challenging because few independent data are generally available for testing or ground-truthing. Uncertainties creep into the estimates due to errors in the inputs, resulting in input-dependent uncertainty, and the subjective nature of the estimation or modeling process, causing process-dependent uncertainty.

More detailed assessments of PAR of *P. falciparum *malaria variation were possible, however, for three African countries where data on census counts or official population estimates were reported at a higher administrative-unit level than those used in the construction of each of the four gridded population datasets: Mali, Namibia, and Tanzania. Data on population counts from the 2009 Mali census at commune level (administrative level 3) were obtained from the Institut National de la Statistique du Mali and matched to administrative-unit data from the Global Administrative Areas Project (http://www.gadm.org). The global population datasets used cercle-level (administrative level 2) data for Mali. For Namibia, 2001 census data matched to enumeration area (administrative level 4) boundaries were obtained from the Namibian Ministry of Health and Social Services and were substantially more detailed than the constituency level (administrative level 2) data used in the construction of the LandScan, GPW, GRUMP, and UNEP datasets. Finally, 2002 census data at ward level (administrative unit level 3) for Tanzania were downloaded from the International Livestock Research Institute (http://64.95.130.4/gis/search.asp?id=442), a level finer than that used in the construction of the global population datasets. Additional file 1, Figure S3 shows the administrative boundaries of the census data for each of the three countries.

Each country spans two or more *P. falciparum *transmission classes (Additional file 1, Figure S3), providing a good test of how each existing dataset had quantified PAR in a range of transmission settings and between classes. Moreover, both the input census or estimate data used in construction of the existing population datasets and the data for assessment for the three countries cover a wide range of administrative levels and average spatial resolutions (ASRs).

For each country, the detailed population data were projected forward to 2007 to match the malaria data, using the same growth rates described in the previous section. PAR estimates from the census data were then calculated by overlaying the *P. falciparum *malaria class map onto the detailed census data and calculating the proportion of each class covering each unit. Populations were assigned to each class based on these proportions. Given the small size of the units in most of the detailed census data, the vast majority of units belonged wholly to one class. The resulting PAR estimates represented refined estimates of PAR for each of the three countries that could be compared to those derived from GRUMP, GPW, LandScan, and UNEP. These comparisons were undertaken through calculation of root mean square errors (RMSEs) between the per-unit PARs in the fine-resolution datasets and those estimated by the four spatial population datasets. As in the previous section, analyses were undertaken on the three datasets both adjusted to common national totals [22] and those left unadjusted.

## Results

### Estimates of national population totals

**Figure 4:**
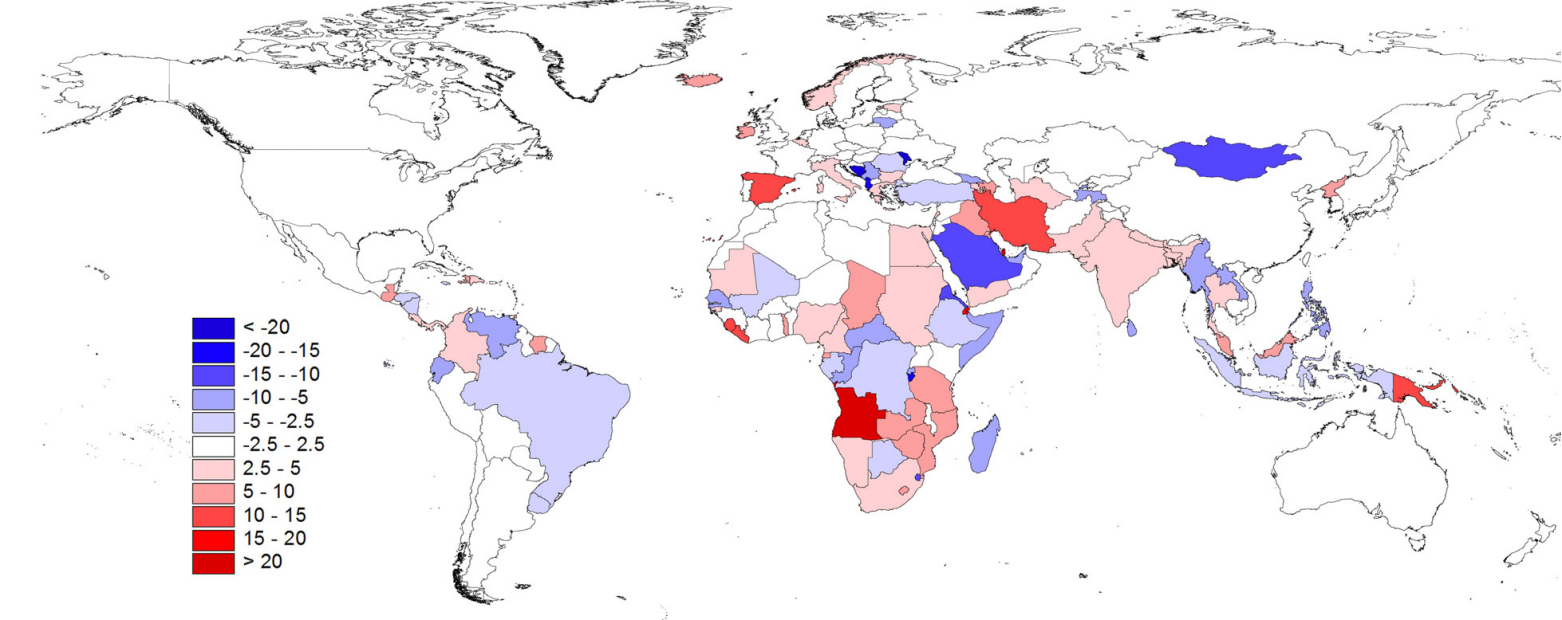
**The differences between national population totals for 2010 estimated by the UNPD and the CIA**. The differences are shown as a percentage change from national population totals estimated by the UNPD.

The results of comparing national population totals estimated by the UNPD (as used for GPW, GRUMP, and UNEP GRID) with those estimated by the CIA (as used in LandScan) are outlined in Figure 4. The map shows the relative effects on population totals, in percentage terms, of changing from a population dataset adjusted to UNPD totals to one adjusted to CIA totals. The differences that can result from such adjustments are evident when considering the extreme case of Angola, where the UNPD estimates a total 2010 population of 18,993,000, while the CIA estimates just 13,068,161, a reduction of 31%. Elsewhere, differences are smaller, but a large number of countries show absolute differences of greater than 5%. Moreover, a clear pattern is evident, with estimates for low-income countries, particularly those in sub-Saharan Africa, varying by greater amounts than for the higher-income regions. For countries defined as "least developed" [22], the average absolute difference is 6.2%, which is significantly different (p < 0.05) from the average absolute difference of 4.3% for the remaining countries.

### Variations in *P. falciparum* PAR

**Figure 5:**
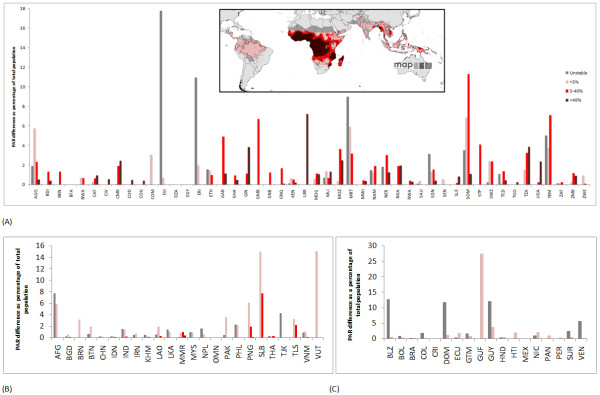
**Variations in estimates of population at risk of *P. falciparum *achievable using LandScan and GRUMP**. Here, the LandScan and GRUMP datasets were adjusted to ensure that national population totals matched those provided by the UN [22]. The difference in PAR estimates are presented as a percentage of total national population (UN estimates), and shown for (A) Africa+, (B) CSE Asia, and (C) the Americas. The ISO country abbreviation for country name is used (http://www.iso.org/iso/english_country_names_and_code_elements).

**Figure 6:**
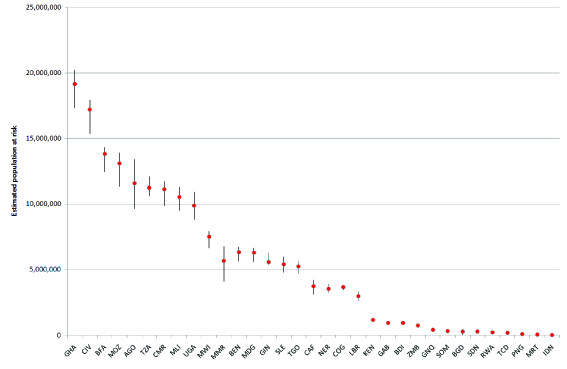
**Plot showing the range of estimates of PAR achievable using four different population datasets**. The plot shows the mean (red circle) and range (black bar) of estimates of populations at risk of *Pf*PR >40% in 2007 achievable using the four population datasets outlined in Table 1. Nigeria (Maximum: 117,672,000, Mean: 112,564,250, Minimum: 101,283,000) and the Democratic Republic of the Congo (Maximum: 56,767,900, Mean: 52,775,275, Minimum: 44,555,900) are not shown here, as their PARs are significantly larger than the other countries. The ISO country abbreviation for country name is used (http://www.iso.org/iso/english_country_names_and_code_elements).

At global and continental scales, Table 3 shows that the choice of population dataset makes only relatively small differences in the estimated proportions at risk, with GRUMP and LandScan estimating roughly similar numbers (Additional file 1, Table S2 shows the estimated numbers at risk using all four population datasets, and Additional file 1, Table S3 shows concordance correlation coefficients [33] for the per-country PAR estimates made by each of the four datasets). However, these estimates mask the much more substantial country-scale variations. Figure 5 summarizes these relative variations (in percentage terms for comparability) in national *P. falciparum *PAR using the two most widely used population datasets in disease studies today, LandScan and GRUMP, adjusted to common national totals. Additional file 1, Figure S2 shows the results for the unadjusted analyses, and there were few differences from Figure 5 because a linear adjustment of population totals results in minimal effects on proportions of the total population residing in different transmission zones. The largest percentage differences occur for the smallest countries, as expected, as relatively small differences in PAR translate to large percentage differences in these cases. Many larger countries, especially in sub-Saharan Africa, also display differences in PAR estimates for certain classes of near to or greater than 5%. These include Angola, Gabon, Liberia, Mozambique, Mauritania, Somalia, Tanzania, and Yemen. Moreover, though relative differences in PAR achievable through switching between LandScan and GRUMP for a large country such as Nigeria are only about 2% for the two transmission classes covering the country, in absolute terms, this translates to differences of more than 3 million people. Figure 6 plots these differences in absolute terms for the *Pf*PR >40% class, through using all four population datasets described in Table 1 and unadjusted to common national totals to highlight the kinds of variations that past studies (Table 2) would have achieved through considering alternative population datasets. For clarity, Nigeria and the Democratic Republic of the Congo are not shown, but the graph highlights again how estimates of those residing in the highest *P. falciparum *transmission zones differ by many millions for countries with the highest numbers at risk.

**Table 3:** Total estimated populations at risk (PAR) of *P. falciparum *malaria in each class by region and in total for the GRUMP and LandScan population datasets.

		PAR (millions)			
		
		Americas	Africa+	CSE Asia	Global
Unstable	LandScan	50.138	18.266	974.086	1042.49
	GRUMP	50.044	21.594	947.371	1019.009

<5%	LandScan	40.312	116.339	601.344	757.995
	GRUMP	40.563	114.313	602.923	757.8

5-40%	LandScan	NA	193.26	71.504	264.764
	GRUMP	NA	197.349	75.214	272.563

>40%	LandScan	NA	350.644	6.124	356.767
	GRUMP	NA	346.607	6.712	353.319

GRUMP = Global Rural Urban Mapping Project

### National-level assessments of PAR estimates

Results of the adjusted national-level assessments in Table 4 suggest that none of the modeling approaches used is consistently more accurate than the others. LandScan or GRUMP, however, which are more recent products and resolved to finer spatial resolutions than GPW and UNEP GRID, were the closest to the fine-resolution PAR estimates in each case. An older, more comprehensive assessment found GRUMP to be a more accurate representation of population distribution for Kenya [18], but in this case, GRUMP and GPW utilized a higher administrative-unit level of census data as input compared to UNEP and LandScan. The results of the analyses on the unadjusted datasets are presented in Additional file 1, Table S4, with few differences from Table 4 because a linear adjustment of population totals results in minimal effects on proportions of the total population residing in different transmission zones.

**Table 4:** Error statistics for comparison of *P. falciparum *populations at risk (PAR) derived from spatial population datasets versus detailed census data.

	LandScan		GPW		GRUMP		UNEP	
	**RMSE**	**CCC**	**RMSE**	**CCC**	**RMSE**	**CCC**	**RMSE**	**CCC**

Mali	83908	0.9997984	128802	0.9995382	**58910**	**0.9999026**	115777	0.999629

Namibia	**24868**	**0.997234**	30581	0.9956934	43900	0.9907193	33615	0.9949944

Tanzania	4340872	0.87304	4106729	0.8929462	**3514209**	**0.9195392**	4718404	0.8530245

Root mean square error (RMSE) and concordance correlation coefficient (CCC) [33] statistics are shown for comparison of *P. falciparum *PAR estimates derived from the four spatial population datasets against the estimates derived from the detailed census data for three countries. The lowest RMSEs and highest CCCs for each country are in bold. Here, the spatial population datasets were adjusted to ensure that national population totals matched those provided by the UN [22]. GPW = Gridded Population of the World, GRUMP = Global Rural Urban Mapping Project, UNEP = United Nations Environment Programme Global Population Databases.

## Discussion

The use of global positioning systems (GPS) and GIS in disease surveys and reporting is becoming increasingly routine, enabling a better understanding of the spatial epidemiology of diseases. In turn, the increased availability of spatially referenced epidemiological data is driving the rapid expansion of disease mapping and spatial modeling methods, which are becoming increasingly detailed and sophisticated, with rigorous handling of uncertainties built in. This expansion has not been matched by advancements in the development of spatial datasets of human population distribution that so often accompany disease maps or spatial models in analyses.

Since the initial development of global spatial population databases in the 1990s, they have enjoyed wide application across multiple fields of research and application [13,34], and in the late 1990s were first applied for estimating populations at risk of disease (Table 2). Since then, the use of spatial population datasets in epidemiological studies has become widespread. Table 2 shows how the different population datasets analyzed here have been used for undertaking similar analyses, yet few studies justify their choice of dataset, and none has assessed the effects of changing to an alternative dataset on results. Results here show that, in the context of an endemic, vector-borne disease, the choice of spatial population dataset can have substantial effects on estimates of populations at risk of disease, particularly for low-income countries where estimates of national population totals are uncertain, census data used in dataset construction are often outdated and of coarse resolution, and national totals are adjusted to differing sizes. Our results also show that assessing which dataset to use remains a difficult task, with tests here showing that none of the datasets was consistently more accurate than others in estimating PAR of *P. falciparum *malaria for the three test countries.

The results presented are focused on the quantification of PAR of *P. falciparum *malaria. However, it is clear that the implications translate to other types of malaria and other endemic, vector-borne diseases, especially those for which spatial population data are already being used to derive population at risk estimates (Table 2). Moreover, as funding for disease mapping continues to grow, the need for accurate spatial population distribution data will also grow if denominator-reliant metrics are required. The effect size of spatial population dataset choice on the outputs of spatial models of directly transmitted disease spread will be a function of the aims of the modeling exercise. However, in any case where spatial population data are used to derive "synthetic populations," for instance in those influenza modeling studies listed in Table 2, there can be no doubt that running such models on the greatly differing distributions in Figure 2 would produce differing epidemiological landscapes and resultant estimated patterns and timings of spread. Calculating metrics on exactly how significant an effect the choice of spatial population dataset used would have on such model predictions is beyond the scope of this article and requires further study. However, the uncertainties inherent in the population datasets are rarely acknowledged and clearly feed into any outputs.

The levels of uncertainty inherent in the sparse disease data used, for instance, to construct maps or parameterize epidemic models may be greater than the uncertainty levels that exist within the spatial population datasets used with them [4,31]. However, the level of uncertainty in the denominator is rarely considered or mentioned. The importance of considering this is underlined by Figures 4, 5 and 6, where, taking the extreme case of Angola, changing from using GRUMP to LandScan produces a relative drop of more than 30% in population size, meaning substantially fewer people at risk of endemic disease or susceptible to emerging diseases. After accounting for this difference, results here show that estimates of PAR of *P. falciparum *malaria for differing transmission classes can change by a further 6%. The uncertainties that exist in estimating total populations residing in some nations likely have substantial implications on estimates of disease risk, burden, and spread, but these go unacknowledged. The difference in estimates of the total population of Angola between the UNPD and the CIA, and the substantial differences for many other low-income countries, highlights that even those nonspatial disease burden estimates reliant on national or per-district denominators [9,35-37] must be cautious and account for uncertainties in the denominator. In many low-income countries, more than 10 years has passed since the last population census (http://unstats.un.org/unsd/demographic/sources/census/censusdates.htm, [15]), and significant uncertainty exists regarding how many people reside in them.

Ideally, a definitive answer to the question of which modeling approach produces superior population distribution mapping accuracy would provide valuable guidance on choosing datasets. Results here, however, show that obtaining this answer is nearly impossible because the most detailed data generally are used in construction of the population datasets, leaving little independent data for testing. Comparisons with the basic assessments undertaken for a few countries where more highly resolved data exist provide inconclusive results. Previous work has suggested that the level of input census data remains an important factor [18] and that detailed mapping of settlements, where the vast majority of people live, can also further improve mapping skill [38]. Deciding among the datasets remains challenging, but the more transparent methodologies, clear documentation of input data, and provision of a mean geographic input unit surface for GPW and GRUMP make those datasets more suited to enabling researchers to understand and quantify the uncertainties inherent in them.

### Improving spatial population dataset construction for epidemiological purposes

Our results highlight that uncertainty in the locations of human populations exists to a varying degree across the world, and that this uncertainty is most pronounced for low-income countries, especially those in sub-Saharan Africa. The advancement of theory, increasing availability of computation, and growing recognition of the importance of robust handling of uncertainty have all contributed to the emergence in recent years of new, sophisticated approaches to the large-scale modeling and mapping of disease. In endemic disease mapping, this has included the use of a special family of generalized linear models known as model-based geostatistics (MBG), generally implemented in a Bayesian framework. These approaches are enabling the explicit quantification of uncertainty associated with disease distributions to be mapped [31], but such approaches have yet to cross over to the demographic databases with which such maps are used. Figures 4, 5, and 6 demonstrate that aspects of the uncertainties inherent in existing population datasets can be quantified. Future work on spatial population datasets should thus focus on integrating such uncertainties into the methods used for their construction as a priority.

As discussed, even when the variations in national total adjustments (Figure 4) are accounted for, substantial variation in PAR estimates deriving from the application of differing modeling methods to coarse-resolution census data are still apparent. Where census datasets are more detailed, the implications of the choice of population distribution modeling approach are reduced. Thus, efforts to improve upon the reliability and precision of spatial population datasets should also focus on obtaining the highest level and most recent census data available. The database behind GPW and GRUMP likely represents the most comprehensive collection of census counts and other official population estimates by administrative unit, and full details are available here: http://sedac.ciesin.columbia.edu/gpw/spreadsheets/GPW3_GRUMP_SummaryInformation_Oct05prod..xls

To identify the priority countries for which both more recent and more detailed population data are required, a simple index through ranking all countries by year of most recent census dataset in the GPW/GRUMP database can be created to highlight those with the oldest data. Further, ranking by population per administrative unit (PPU) highlights those with the coarsest census data. These ranks were then summed for each country, and the top 20 countries in terms of having the oldest and coarsest resolution population data are shown in Table 5 (the top 50 are shown in Additional file 1, Table S5). All the countries listed are either in Africa or Asia, with the individual columns showing that population count data from the 1980s, and at a spatial resolution where on average more than 1 million people reside in each administrative unit, are still being used to estimate diseases risks, burdens, spread, and dynamics.

**Table 5:** The top 20 priority countries in terms of spatially referenced population data needs

Rank	Country	PPU	Population data year
1	Iraq	1,258,000	1985
2	Congo, Democratic Republic of the	347,000	1984
3	Chad	527,000	1990
4	Syria	1,241,000	1994
5	Libya	223,000	1984
6	Cameroon	255,000	1987
7	Sudan	358,000	1993
8	Papua New Guinea	241,000	1990
9	United Arab Emirates	399,000	1995
10	Nigeria	231,000	1991
11	Togo	216,000	1991
12	Pakistan	1,309,000	1998
13	Egypt	281,000	1996
14	Iran	260,000	1996
15	Bhutan	110,000	1985
16	Algeria	634,000	1998
17	Guinea	257,000	1996
18	Uzbekistan	118,000	1989
19	Eritrea	94,000	1984
20	Senegal	107,000	1985

The ranks are based on ranking all country data in the GPW/GRUMP database (http://sedac.ciesin.columbia.edu/gpw/spreadsheets/GPW3_GRUMP_SummaryInformation_Oct05prod.xls) by population per unit (PPU) and date of the input population count data, then summing these to create a simple combined rank score. Additional file 1, Table S4 shows the top 50 countries.

With the vast majority of human population residing in settlements, on which increasingly accurate, detailed, and reliable datasets are becoming available, the accurate mapping of settlements will improve our abilities to accurately quantify human population distributions. Moreover, those residing in large settlements face differing disease risks [39], and settlements are often used to define patches, nodes, or metapopulations in network-based epidemic models [4]. Efforts to improve both population and settlement spatial data have begun through the launch of a number of projects. The AfriPop project (http://www.afripop.org) aims to provide detailed and freely available population distribution maps for Africa, focusing initially on (i) creating a database of more contemporary and finer resolution census data for sub-Saharan countries, and (ii) mapping settlements across Africa at finer resolution and with greater precision. The population estimation by remote sensing (POPSATER) project (http://www.ulb.ac.be/rech/inventaire/projets/7/PR4417.html) aims to combine remotely sensed data with field survey data to improve population mapping methodologies and create maps of small urban and rural areas in sub-Saharan Africa. Additionally, other projects are focused on improving the mapping of urban areas [40,41] and land cover in general [42,43], providing valuable data for guiding population mapping over large areas [38,44]. All of these projects are, however, disconnected and small in scope, length, and capacity. At a time when the mapping of infectious diseases is garnering increasing donor support, mapping of the denominator remains poorly funded.

Finally, while great advances in our abilities to quantify population distributions over large areas have been made, these have been focused solely on the simple enumeration of total population numbers residing in grid cells. The effects of diseases in terms of morbidity, mortality, and speed of spread and the implications for planning and targeting interventions vary substantially with demographic profiles, with clear risk groups and vulnerable populations existing. Breakdowns of population counts by age and sex are routinely collected during national censuses and maintained in finer detail within microcensus data (https://international.ipums.org/international/). Moreover, demographic surveillance systems (http://www.measuredhs.com/) continue to collect representative and contemporary samples from clusters of communities in low-income countries where census data may be less detailed and not collected regularly. Together, these datasets form a rich resource for quantifying and understanding the spatial variations in the sizes and distributions of those most at risk of disease, yet at present, they remain unconnected data scattered across national statistical offices and websites. At the same time, as calls are being made for improved access to health data [45,46], efforts should be made to gather such demographic datasets into a central resource and better quantify the spatial distributions of vulnerable groups, including infants, children under 5 years old, pregnant women, and the elderly.

## Conclusions

Spatial medical intelligence and disease modeling are becoming central to the effective planning, implementation, monitoring, and evaluation of disease control. Significant advances in the approaches to mapping and modeling of disease risks and epidemic spread have recently been made, supported increasingly by the collection of geospatially referenced survey data. Such advances also involve the incorporation of models of uncertainty in output disease estimates and models, but rarely is the uncertainty inherent in the human population datasets commonly used to provide the denominator even acknowledged. Using the example of *P. falciparum *PAR estimation, we have shown that these uncertainties can significantly impact findings. The quantification of uncertainties inherent in existing spatial population datasets, and the improvement of demographic evidence bases, represents an important research direction if spatial approaches to disease modeling and burden estimation are to become more accurate.

## Competing interests

The authors declare that they have no competing interests.

## Authors' contributions

AJT conceived, designed, and carried out the analysis and wrote the manuscript. NC conducted data analysis. CL and RWS provided and helped interpret data, helped structure and interpret the analyses, and edited the manuscript. PWG helped structure and interpret the analyses, and edited the manuscript. All authors read and approved the final manuscript.

## Supplementary Material

Additional file 1 Tables S1-S5 and Figures S1-S3 Click here for file
