# Supplementary material for: The effects of spatial population dataset choice on estimates of population at risk of disease
Source: Popul Health Metr. 2011 Feb 7;9:4. doi: 10.1186/1478-7954-9-4 (PMC3045911; doi:10.1186/1478-7954-9-4)
Supplement: Additional file 1 — Tables S1-S5 and Figures S1-S3 [file 1478-7954-9-4-S1.PDF]

Supporting Information

Scale	Application	Population map used [Reference]
Africa	PAR *Pf*-hookworm coinfection	GPWv3 [1]
Global	Pregancies at risk of *Pf* and *Pv* malaria	GRUMPα [2]
Global	PAR *Pf*	GRUMPα [3-5]
Global	PAR *Pv*	GRUMPα [5], GRUMPβ [6]
Global	PAR malaria	GPWv2 [7]
Global	Clinical *Pf* burden	GPWv3[8], GRUMPα [9]
Africa	ITN coverage	GRUMPα [10]
Global	Coverage of funding for *Pf* control	GRUMPα [11], GRUMPβ [2]
Global	*Pf* and *Pv* elimination feasibility	GRUMPα [12]
Global	Human migration and *Pf* movements	GRUMPα [13]
Global	Urbanization effects on *Pf* transmission	GPWv3 [14], GRUMPα [15]
National	PAR *Pf* in Zambia	Landscan 2006 [16]
Africa	Future PAR *Pf*	GPWv3 [17]
Global	Effects of climate change on malaria PAR	GPWv2 [18, 19]
Africa	*Pf* risk mapping	GPWv3 [20]
Africa	PAR *Pf*	UNEP [21], GRUMPα [22]
West Africa	PAR *Pf*	UNEP [23]
Global	*Pf* risk mapping	GPWv2 [24], GRUMPα [25]

Table S1. Malaria-related studies that have utilized large area gridded population datasets. GPW = Gridded Population of the World, GRUMP = Global Rural Urban Mapping Project, UNEP = United Nations Environment Programme Global Population Databases, USGS = United States Geological Survey Population datasets.

		PAR		
		Americas	Africa+	CSE Asia
Unstable	Landscan	50,138,167	18,266,064	974,086,156
	GPW3	48,607,543	23,309,881	942,830,207
	GRUMP	50,044,331	21,593,752	947,371,158
	UNEP	38,944,853	13,628,956	703,465,430
<5%	Landscan	40,311,726	116,338,610	601,344,331
	GPW3	40,717,470	106,585,197	593,253,313
	GRUMP	40,563,384	114,313,126	602,923,047
	UNEP	35,948,739	65,910,766	384,360,353
5-40%	Landscan	NA	193,260,281	71,504,201
	GPW3	NA	185,111,034	75,453,864
	GRUMP	NA	197,349,050	75,213,946
	UNEP	NA	147,850,121	42,871,904
>40%	Landscan	NA	350,643,756	6,123,664
	GPW3	NA	354,000,328	7,321,183
	GRUMP	NA	346,607,237	6,711,505
	UNEP	NA	302,919,397	4,154,098

Table S2. Total estimated populations at risk (PAR) of *P. falciparum* in each class by region and in total for each population dataset. GPW = Gridded Population of the World, GRUMP = Global Rural Urban Mapping Project, UNEP = United Nations Environment Programme Global Population Databases.

	LandScan	GPW	GRUMP	UNEP
LandScan	X	0.999096	0.9992544	0.9541364
GPW		X	0.9999828	0.9647
GRUMP			X	0.9636067
UNEP				X

A

	LandScan	GPW	GRUMP	UNEP
LandScan	X	0.9994204	0.9992751	0.9334398
GPW		X	0.9998924	0.9284618
GRUMP			X	0.924434
UNEP				X

B

	LandScan	GPW	GRUMP	UNEP
LandScan	X	0.9923876	0.9938765	0.8763075
GPW		X	0.9920221	0.8730398
GRUMP			X	0.8570616
UNEP				X

C

	LandScan	GPW	GRUMP	UNEP
LandScan	X	0.9923876	0.9938765	0.8763075
GPW		X	0.9920221	0.8730398
GRUMP			X	0.8570616
UNEP				X

D

Table S3. Concordance correlation coefficients for per-country PAR estimates made by each of the four population datasets for A: Unstable risk, B: *Pf*PR$_{2-10}$<5%, C: *Pf*PR$_{2-10}$ = 5-40%, D: *Pf*PR$_{2-10}$ > 40%. The values show the strength of correlation between estimated PARs when comparing different spatial population datasets.

	Landscan	GPW	GRUMP	UNEP
Namibia	**22314**	63380	62609	103804
Tanzania	4351628	4095408	**3335634**	5319315
Mali	717699	137853	**100356**	489515

Table S4. Error statistics for comparison of *P. falciparum* populations at risk (PAR) derived from spatial population datasets versus detailed census data. Root mean square error (RMSE) statistics are shown for comparison of *P. falciparum* PAR estimates derived from the four spatial population datasets against the estimates derived from the detailed census data for three countries. The lowest RMSEs for each country are in bold text. Here, each of the datasets were not adjusted to common national totals (in contrast to Table 3 in the main document). GPW = Gridded Population of the World, GRUMP = Global Rural Urban Mapping Project, UNEP = United Nations Environment Programme Global Population Databases.

Rank	Country	PPU	Census data year
1	Iraq	1258	1985
2	Congo, Democratic Republic	347	1984
3	Chad	527	1990
4	Syrian Arab Republic	1241	1994
5	Libyan Arab Jamahiriya	223	1984
6	Cameroon	255	1987
7	Sudan	358	1993
8	Papua New Guinea	241	1990
9	United Arab Emirates	399	1995
10	Nigeria	231	1991
11	Togo	216	1991
12	Pakistan	1309	1998
13	Egypt	281	1996
14	Iran	260	1996
15	Bhutan	110	1985
16	Algeria	634	1998
17	Guinea	257	1996
18	Uzbekistan	118	1989
19	Eritrea	94	1984
20	Senegal	107	1985
21	Tajikistan	99	1989
22	Lesotho	209	1996
23	Azerbaijan	108	1990
24	Saudi Arabia	1604	2000
25	Turkmenistan	86	1989
26	Swaziland	247	1997
27	Uruguay	171	1996
28	Liberia	70	1983
29	Ethiopia	119	1994
30	Turkey	848	2000
31	China	523	2000
32	Guyana	79	1991
33	Central African Republic	69	1988
34	Lebanon	128	1996
35	Myanmar	165	1997
36	Benin	80	1992
37	Kazakhstan	62	1989
38	Djibouti	128	1998
39	Rwanda	55	1991
40	Serbia and Montenegro	2658	2001
41	Ivory Coast	89	1998
42	East Timor	46	1990
43	Republic of Moldova	88	1998
44	Zambia	173	2000
45	Bosnia-Herzegovina	1301	2001
46	Ghana	172	2000
47	Kyrgyz Republic	89	1999
48	Congo	60	1996

49	Belarus	84	1999
50	Somalia	110	2000

Table S5. The top 50 priority countries in terms of spatially-referenced population data needs. The ranks are based on ranking all country data in the GPW/GRUMP database (http://sedac.ciesin.columbia.edu/gpw/spreadsheets/GPW3_GRUMP_SummaryInformation_Oct05prod.xls) by population per unit (PPU) and date of the input population count data, then summing these to create a simple combined rank score.

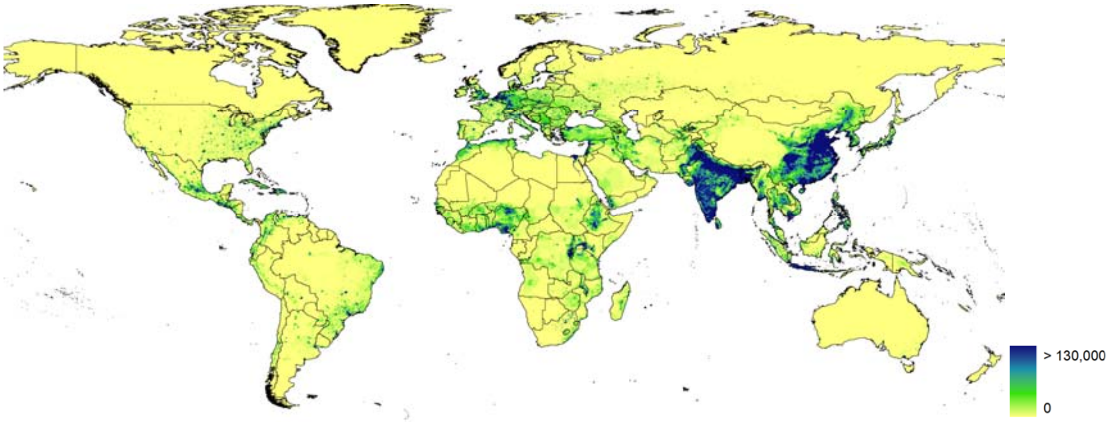

A

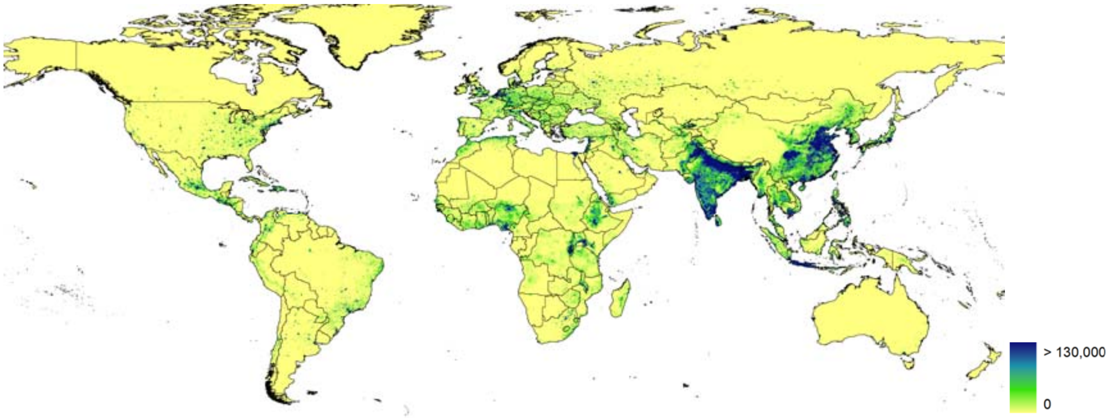

B

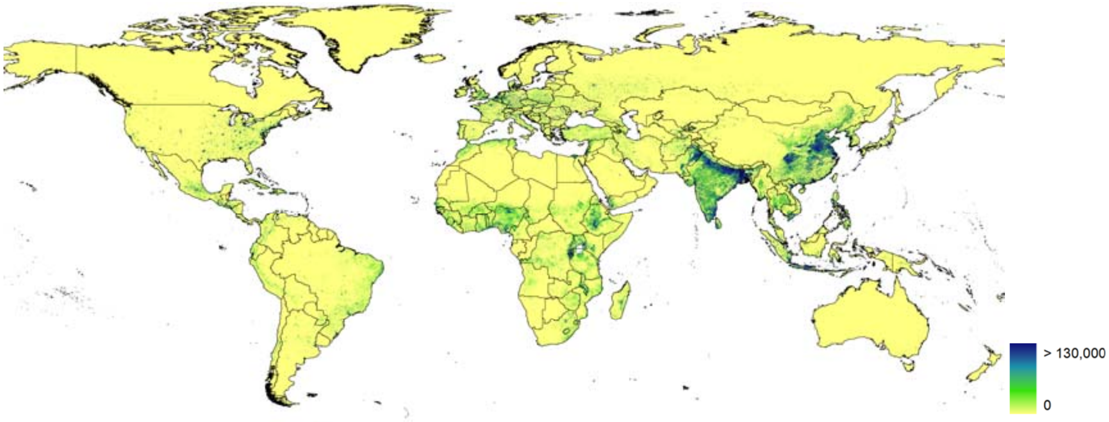

C

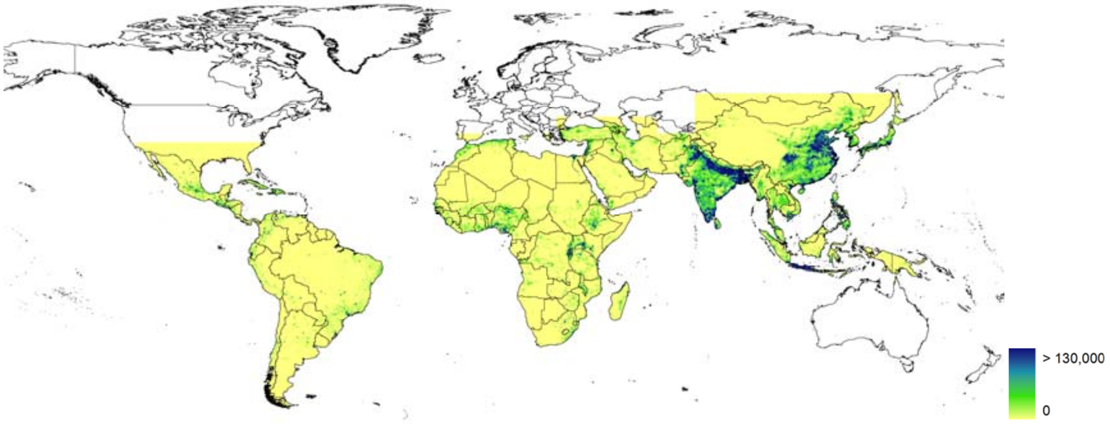

D

Figure S1. The four spatial population datasets analysed for this study. The datasets are: (a) Gridded Population of the World (GPW) version 3, (b) the Global Rural Urban Mapping Project (GRUMP) alpha version, (c) LandScan 2008 and (d) UNEP Grid. Details on each dataset can be found in Table 1 of the main manuscript.

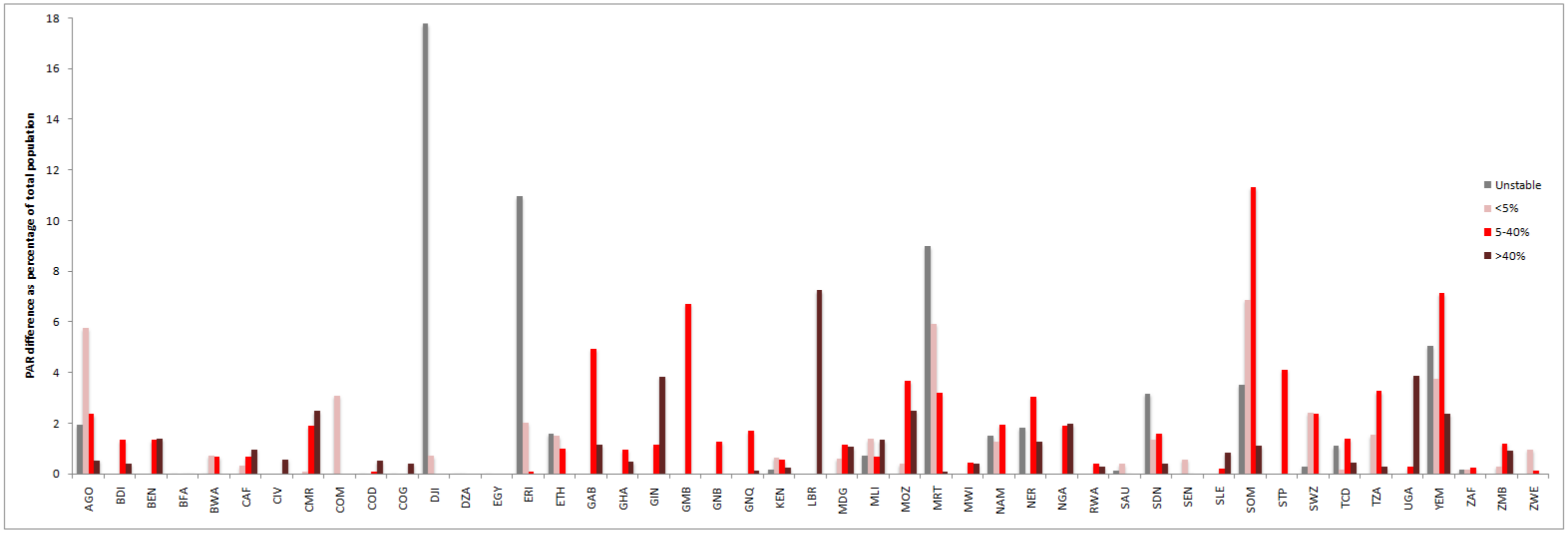
PAR difference as percentage of total population
Unstable
<5%
5-40%
>40%
AGO BDI BEN BFA BWA CAF CIV CMR COM COD COG DJI DZA EGY ERI ETH GAB GHA GIN GMB GNB GNQ KEN LBR MDG MLI MOZ MRT MWI NAM NER NGA RWA SAU SDN SEN SLE SOM STP SWZ TCD TZA UGA YEM ZAF ZMB ZWE

A

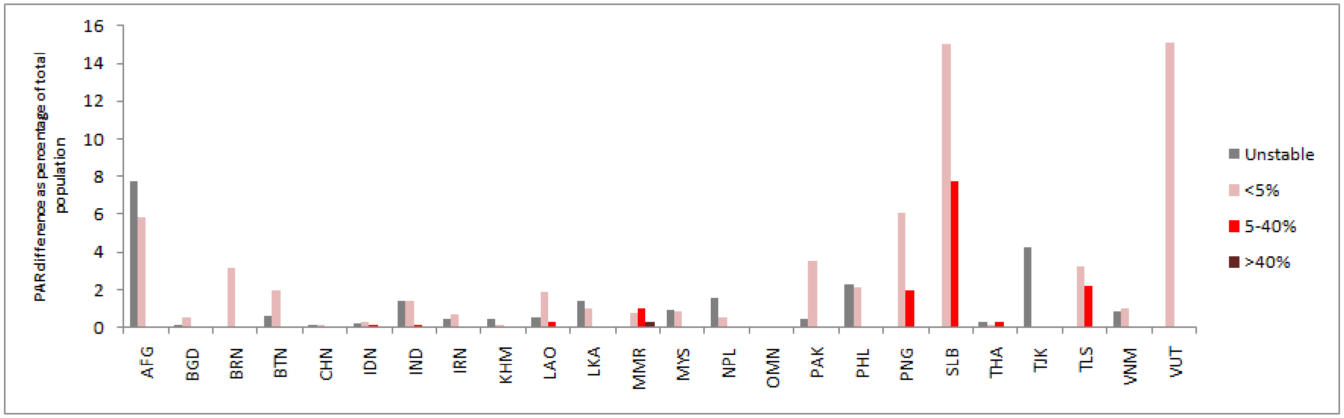
PAR difference as percentage of total population
Unstable
<5%
5-40%
>40%
AFG BGD BRN BTN CHN IDN IND IRN KHM LAO LKA MMR MYS NPL OMN PAK PHL PNG SLB THA TJK TLS VNM VUT

B

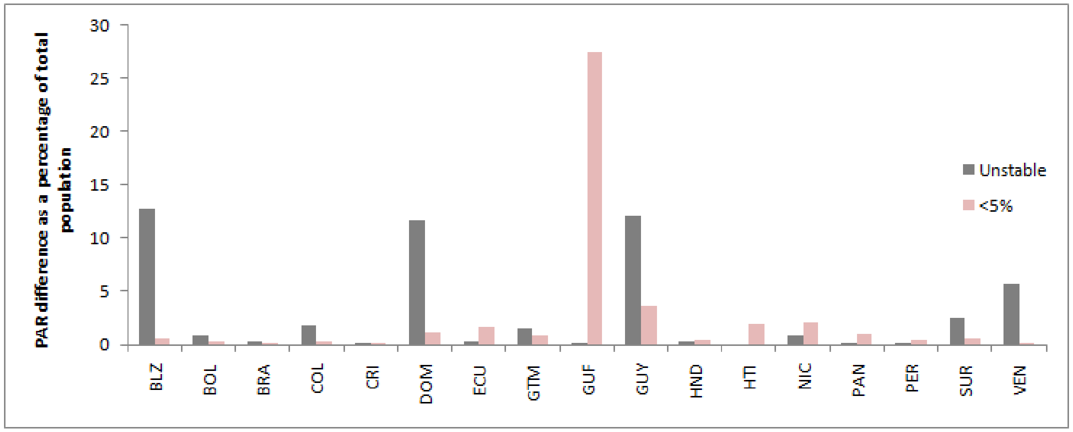

C

Figure S2. Variations in estimates of population at risk of *P. falciparum* achievable using LandScan and GRUMP. Here, the LandScan and GRUMP datasets were not adjusted to common national totals (in contrast to Figure 2 of the main document). The estimates are presented as a percentage of total national population (UN estimates), and shown for (i) Africa+, (ii) CSE Asia and (iii) the Americas. The ISO country abbreviation for country name is used (http://www.iso.org/iso/english_country_names_and_code_elements).

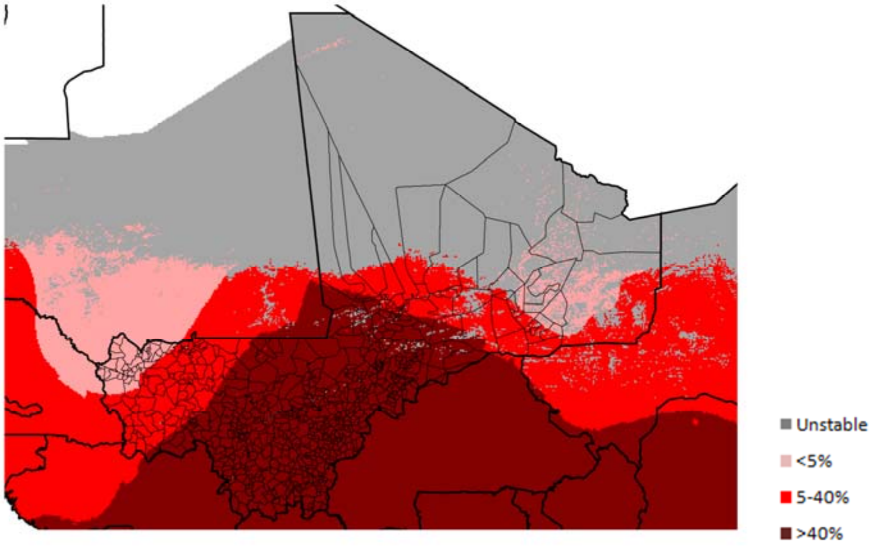

A

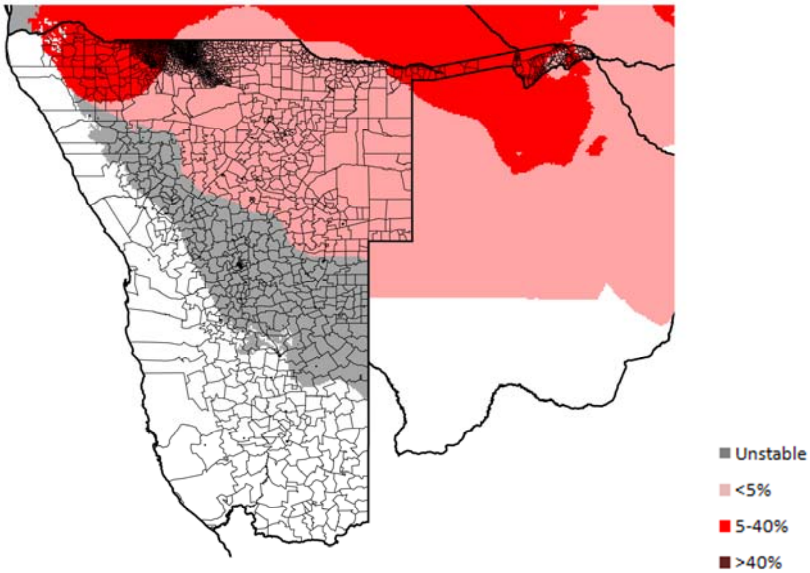

B

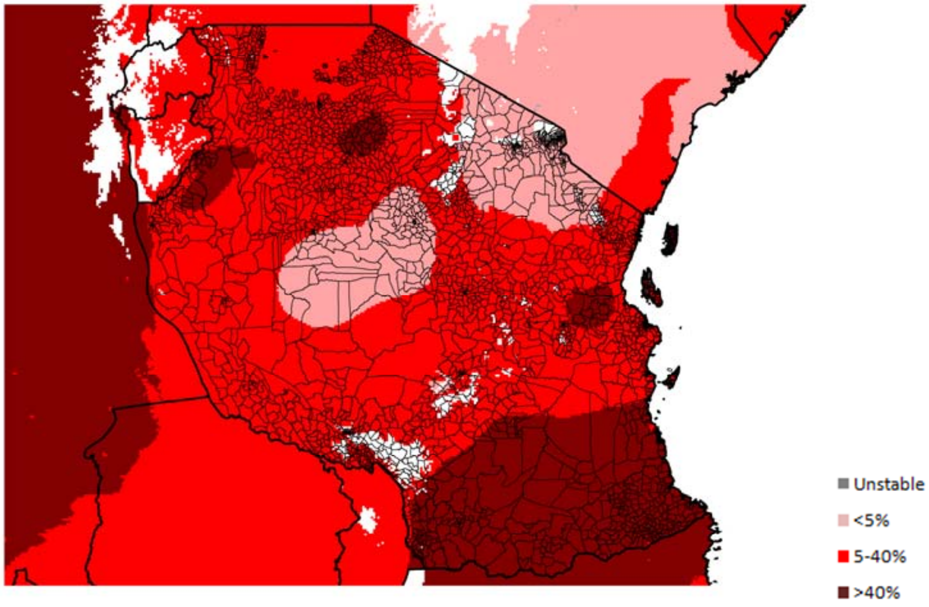

C

Figure S3. Administrative unit boundaries of the census data used to test the accuracy of the global population datasets. The figure shows the administrative unit boundaries of the census data used for (a) Mali, (b) Namibia and (c) Tanzania, overlaid onto the predicted *P. falciparum* malaria *Pf*PR$_{2\text{-}10}$ endemicity classes. They are categorized as low risk *Pf*PR$_{2\text{-}10}$ < 5%, light red; intermediate risk *Pf*PR$_{2\text{-}10}$ = 5% to 40%, medium red; and high risk *Pf*PR$_{2\text{-}10}$ > 40%, dark red. The map shows the class to which *Pf*PR$_{2\text{-}10}$ has the highest predicted probability of membership. The rest of the land area was defined as unstable risk (medium grey areas, where *Pf*API = 0.1 per 1,000 pa) or no risk (light grey).

References

1. Brooker SJ, Clements ACA, Hotez PJ, Hay SI, Tatem AJ, Bundy DAP, Snow RW: **The co-distribution of *Plasmodium falciparum* and hookworm among African schoolchildren.** *Malaria Journal* 2006, **5:**99.
2. Dellicour S, Tatem AJ, Guerra CA, Snow RW, ter Kuile FO: **Quantifying the number of pregnancies at risk of malaria in 2007: a demographic study.** *PLoS Medicine* 2010, **7:**e1000221.
3. Guerra CA, Gikandi PW, Tatem AJ, Noor AM, Smith DL, Hay SI, Snow RW: **The limits and intensity of *Plasmodium falciparum* transmission: implications for malaria control and elimination worldwide.** *PLoS Medicine* 2008, **5:**e38.
4. Hay SI, Guerra CA, Gething PW, Patil AP, Tatem AJ, Noor AM, Kabaria CW, Manh BH, Elyazar IRF, Brooker SJ, et al: **World malaria map: *Plasmodium falciparum* endemicity in 2007.** *PLoS Medicine* 2009, **6:**e1000048.
5. Guerra CA, Snow RW, Hay SI: **Mapping the global extent of malaria in 2005.** *Trends in Parasitology* 2006, **22:**353-358.
6. Guerra CA, Howes RE, Patil AP, Gething PW, Van Boeckel TP, Temperley WH, Kabaria CW, Tatem AJ, Manh BH, Elyazar IRF, et al: **The international limits and population at risk of *Plasmodium vivax* transmission in 2009.** *PLoS Neglected Tropical Diseases* 2010, **4**:e774.
7. Hay SI, Guerra CA, Tatem AJ, Noor AM, Snow RW: **The global distribution and population at risk of malaria: past, present, and future.** *Lancet Infectious Diseases* 2004, **4:**327-336.
8. Snow RW, Guerra CA, Noor AM, Myint HY, Hay SI: **The global distribution of clinical episodes of *Plasmodium falciparum* malaria.** *Nature* 2005, **434:**214-217.
9. Hay SI, Okiro EA, Gething PW, Patil AP, Tatem AJ, Guerra CA, Snow RW: **Estimating the global clinical burden of *Plasmodium falciparum* malaria in 2007.** *PLoS Medicine* 2010, **7:**e100029.
10. Noor AM, Mutheu JJ, Tatem AJ, Hay SI, Snow RW: **Insecticide-treated net coverage in Africa: mapping progress in 2000-07.** *The Lancet* 2008, **373:**58-67.
11. Snow RW, Guerra CA, Mutheu JJ, Hay SI: **International funding for malaria control in relation to populations at risk of stable *Plasmodium falciparum* transmission.** *PLoS Medicine* 2008, **5:**e142.
12. Tatem AJ, Smith DL, Gething PW, Kabaria CW, Snow RW, Hay SI: **Ranking elimination feasibility among malaria endemic countries.** *The Lancet* 2010, **376:**1579-1591.
13. Tatem AJ, Smith DL: **International population movements and regional *Plasmodium falciparum* malaria elimination strategies.** *Proceedings of the National Academy of Sciences* 2010, **in press**.
14. Hay SI, Guerra CA, Tatem AJ, Atkinson PM, Snow RW: **Urbanization, malaria transmission and disease burden in Africa.** *Nature Reviews Microbiology* 2005, **3:**81-90.
15. Tatem AJ, Guerra CA, Kabaria CW, Noor AM, Hay SI: **Human population, urban settlement patterns and their impact on *Plasmodium falciparum* malaria endemicity.** *Malaria Journal* 2008, **7:**218.
16. Riedel N, Vounatsou P, Miller JM, Gosoniu L, Chizema-Kawesha E, Mukonka V, Steketee RW: **Geographical patterns and predictors of malaria risk in Zambia: Bayesian geostatistical modelling of the 2006 Zambia national malaria indicator survey (ZMIS).** *Malaria Journal* 2010, **9:**37.
17. Hay SI, Tatem AJ, Guerra CA, Snow RW: **Foresight on population at malaria risk in Africa: 2005, 2015 and 2030: Scenario review paper prepared for the Detection and Identification of Infectious Diseases Project (DIID), Foresight Project, Office of Science and Technology, London, UK.** In *Book Foresight on population at malaria risk in Africa: 2005, 2015 and 2030: Scenario review paper prepared for the Detection and Identification of Infectious Diseases Project (DIID), Foresight Project, Office of Science and Technology, London, UK*. pp. Pg. 40. City; 2006:Pg. 40.

18. van Lieshout M, Kovats RS, Livermore MTJ, Martens P: **Climate change and malaria: analysis of the SRES climate and socio-economic scenarios.** *Global Environmental Change* 2004, **14:**87-99.
19. Rogers DJ, Randolph SE: **The global spread of malaria in a future, warmer world.** *Science* 2000, **289:**1763-1766.
20. Moffett A, Shackelford N, Sarkar S: **Malaria in Africa: Vector species' niche models and relative risk maps.** *PLoS ONE* 2007, **2:**e824.
21. Snow RW, Craig M, Deichmann U, Marsh K: **Estimating mortality, morbidity and disability due to malaria among Africa's non-pregnant population.** *Bulletin of the World Health Organization* 1999, **77:**624-640.
22. Teklehaimanot A, McCord G, Sachs J: **Scaling up malaria control in Africa: an economic and epidemiological assessment.** *American Journal of Tropical Medicine and Hygiene* 2007, **77:**138-144.
23. Gemperli A, Sogoba N, Fondjo E, Mabaso M, Bagayoko M, Briet OJT, Anderegg D, Liebe J, Smith T, Vounatsou P: **Mapping malaria transmission in West and Central Africa.** *Tropical Medicine and International Health* 2006, **11:**1032-1046.
24. Kiszewski A, Mellinger A, Spielman A, Malaney P, Sachs SE, Sachs J: **A global index representing the stability of malaria transmission.** *American Journal of Tropical Medicine and Hygiene* 2004, **70:**486-498.
25. Guerra CA, Snow RW, Hay SI: **Determining the global spatial limits of malaria transmission in 2005.** *Advances in Parasitology* 2006, **62:**157-179.
